# Updates on Emerging Interventions for Autosomal Recessive *ABCA4*-Associated Stargardt Disease

**DOI:** 10.3390/jcm12196229

**Published:** 2023-09-27

**Authors:** Liang Wang, Serena M. Shah, Simran Mangwani-Mordani, Ninel Z. Gregori

**Affiliations:** 1 University of Miami Miller School of Medicine, Miami, FL 33136, USA; liang.wang@med.miami.edu (L.W.); serenamshah@miami.edu (S.M.S.); 2 Department of Ophthalmology, Bascom Palmer Eye Institute, University of Miami Miller School of Medicine, Miami, FL 33136, USA; simranmangwanim@gmail.com; 3 Miami Veterans Administration Medical Center, Miami, FL 33125, USA

**Keywords:** Stargardt disease, *ABCA4*, gene therapy, visual cycle modulators, stem cell therapy, adeno-associated viral vectors, deuterated vitamin A, emixustat, human embryonic stem cells

## Abstract

Autosomal recessive Stargardt disease (STGD1) is an inherited retinal degenerative disease associated with a mutated ATP-binding cassette, subfamily A, member 4 (*ABCA4*) gene. STGD1 is the most common form of juvenile macular degeneration with onset in late childhood to early or middle adulthood and causes progressive, irreversible visual impairment and blindness. No effective treatment is currently available. In the present article, we review the most recent updates in clinical trials targeting the management of STGD1, including gene therapy, small molecule therapy, and stem cell therapy. In gene therapy, dual adeno-associated virus and non-viral vectors have been successful in delivering the human *ABCA4* gene in preclinical studies. For pharmaceutical therapies ALK-001, deuterated vitamin A shows promise with preliminary data for phase 2 trial, demonstrating a decreased atrophy growth rate after two years. Stem cell therapy using human pluripotent stem cell-derived retinal pigment epithelium cells demonstrated long-term safety three years after implantation and visual acuity improvements in the first two years after initiation of therapy. Many other treatment options have ongoing investigations and clinical trials. While multiple potential interventions have shown promise in attenuating disease progression, further exploration is necessary to demonstrate treatment safety and efficacy.

## 1. Introduction

Autosomal recessive Stargardt disease (STGD1, OMIM#248200) is an inherited macular dystrophy also known as fundus flavimaculatus (the variant with more widespread flecks and less macular involvement), associated with disease-causing genetic variants in the ATP-binding cassette, sub-family A, member 4 (*ABCA4)* gene [1]. It is one of the most common inherited retinal diseases with a prevalence estimated at 1 in 8000–10,000 [2]. The carrier frequency is as high as 1 in 20 in some populations. The disorder is characterized by progressive central vision loss and may be accompanied by peripheral vision loss in later stages or more severe cases of the disease [3,4]. STGD1 commonly manifests during childhood or early adulthood, and less frequently in later adulthood, when later onset confers a better visual prognosis with a slower rate of visual loss [5]. The classical clinical features include variable pigmentary changes and pisciform flecks in the macula progressing to a variable extent of chorioretinal atrophy, which depends on the severity of the underlying pathogenic *ABCA4* variants, with more severe variants seen in childhood-onset STGD1 [4]. Over 2000 variants within the *ABCA4* gene have been described [6]. Patients report decreased visual acuity, central scotomas, and color vision abnormalities, with variable degrees of nyctalopia and visual field constriction in later stages or more severe cases. Diagnosis involves ophthalmic evaluation, multimodal imaging, and genetic testing [7]. The *ABCA4* gene encodes a transmembrane protein, a member of the ATP-binding cassette transporter superfamily, sub-family A, member 4, which plays an essential role in the retinoid recycling in the visual cycle and localizes to the disc membranes in the outer segments of rods and cones. Lack of ABCA4 protein leads to accumulation of visual cycle byproducts (lipofuscin) and dysfunction of the retinal pigment epithelium (RPE) and subsequent photoreceptor degeneration [8,9]. Although no cure currently exists, promising treatment strategies including genetic therapy, pharmacological treatments, and stem cell therapy, are being explored. Advancing our understanding of the underlying mechanisms and developing effective interventions are essential for managing this debilitating retinal disorder and preventing irreversible vision loss.

### 1.1. Clinical Presentation and Diagnostic Testing

**Figure 1:**
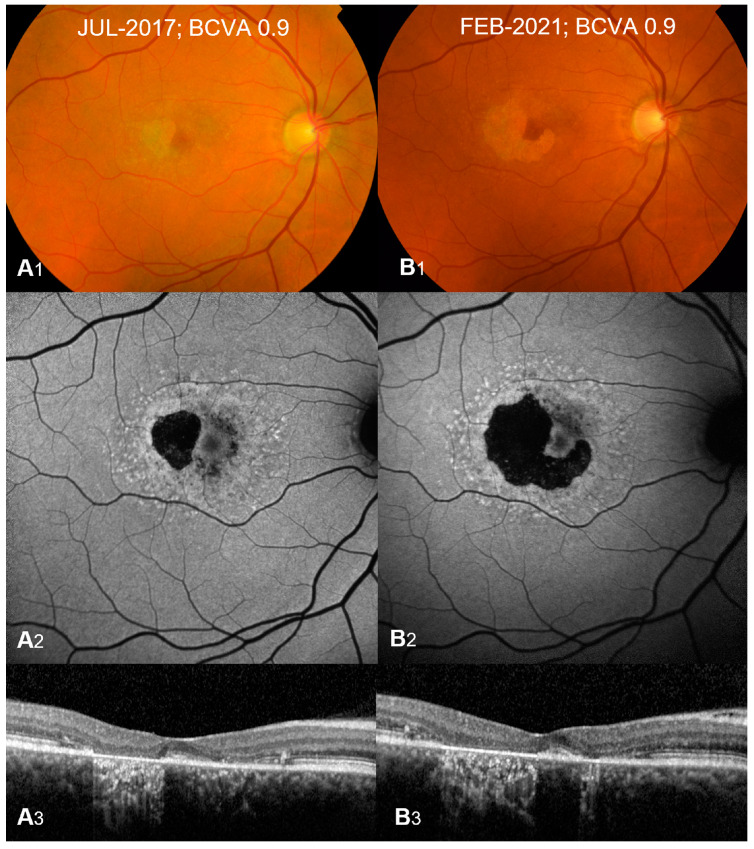
Typical presentation of Stargardt Type 1 at diagnosis (**A_1_**–**A_3_**) and 3-year follow-up (**B_1_–B_3_**). Color fundus (**A_1_**,**B_1_**) shows macular atrophy with yellow-white retinal flecks. Fundus autofluorescence (**A_2_**,**B_2_**) shows patches of hypoautofluorescence surrounded by an increased signal with flecks of both increased and decreased autofluorescence. Optical coherence tomography B-scans (**A_3_**,**B_3_**) show outer retinal and retinal pigment epithelium loss with hypertransmission defects corresponding to atrophy. Atrophy growth is significant at 3-year follow-up (**B_1_**–**B_3_**) compared to presentation at diagnosis (**A_1_**–**A_3_**). Best corrected visual acuity (BCVA) remains stable at 0.9 Snellen decimals (20/160). Reprinted from [1], used under an open-access license agreement distributed under the terms of the Creative Commons CC-BY license.

STGD1 presents with a spectrum of clinical features. On initial ophthalmoscopy, many children with STGD1 can have a normal-appearing fundus with or without vision loss; therefore, diagnosis can be delayed until the appearance of flecks or visual symptoms becomes significant enough to warrant deeper investigation with multimodal imaging including a combination of fundus fluorescein autofluorescence (FAF), optical coherence tomography (OCT), and electroretinography (ERG) [4,10,11,12,13]. Very young children may also present with symmetrical yellowish fine dots in the central macula. Later on, the characteristic key clinical features appear and include bilateral, progressive central vision loss, which encompasses both dyschromatopsia and central scotomata, macular atrophy, and yellow-white flecks within the RPE at the posterior pole [3]. The yellow-white flecks (Figure 1) represent the accumulation of lipofuscin, a yellowish autofluorescent pigment in the RPE cells that blocks the underlying choroidal fluorescence and causes the classic dark appearance of the choroid on FAF in 65–86% of patients [10,11].

FAF is a useful non-invasive imaging modality. It may show an increased signal from excessive lipofuscin and highlights the flecks with both increased and decreased autofluorescence in some patients and reduced central autofluorescence surrounded by a brighter signal similar to a bull’s eye appearance [1,10] (Figure 1). Areas of a decreased FAF signal may appear later and correspond to areas of RPE atrophy with secondary photoreceptor loss. A significant correlation has been shown between FAF subtype and genotype, with milder pathological variants producing a localized low signal at the fovea with a surrounding homogeneous background, whereas more severe variants (including nonsense) correlate with areas of hypofluorescent atrophic areas [10].

OCT typically shows outer retinal loss in the central macula (Figure 1) with possible peripapillary sparing of the retina and RPE, which if present strongly supports the diagnosis of autosomal recessive STGD1 and may also show the earliest abnormality in young children consisting of external limiting membrane thickening [10,12,14].

Interestingly, in a study conducted by Bax et al., it was reported that 11.1% of a cohort of 280 STGD1 patients with a median age of 8 years (range, 1–18 years) had no fundus abnormalities, which resulted in a median delay in diagnosis of 3 years when the first fundus abnormalities appeared and included central RPE alterations most frequently without flecks (43%), bull’s eye maculopathy (33%), and/or parafoveal flecks (24%). These patients were commonly misdiagnosed with other ocular and non-ocular conditions, such as amblyopia, myopia, optic disk pathology, mental health problems, tension headache, bulbar neuritis, and uveitis [11]. Once imaging has been completed, results of patients with STGD1 typically show loss of normal architecture at the central macula and reduced central autofluorescence surrounded by an increased signal or a bull’s eye maculopathy on FAF (Figure 1) [10].

ERG has been shown to correlate with the severity of the disease and helps to establish visual prognosis for the patients [13]. Group 1 demonstrates a severe pattern ERG due to macular dysfunction with normal full-filed ERG (ff-ERG); Group 2 shows generalized cone function loss on ff-ERG; and Group 3 shows both cone and rod function loss on ff-ERG. These groups do not represent stages of the disease but have prognostic implications, with all patients in Group 3 undergoing clinically significant worsening as opposed to only 20% in Group 1 showing clinically significant progression [13].

Most patients experience visual symptom onset during childhood, but some experience it as young adults or even in late adulthood. Childhood disease manifestation usually affects children aged 6–12 years old and causes a rapid, severe decline in visual acuity over the consequential 12–24 months [5]. Later symptom onset has been associated with better visual acuity prognosis, most likely due to the increased presence of missense variants causing foveal-sparing disease and decreased presence of nonsense mutations causing deleterious variants [5]. Overall, the age of onset and rate of disease progression are diverse, but most patients experience symptoms by the time they are in their teenage years, and almost all patients become severely visually impaired or legally blind by the fourth to seventh decade of life [15]. A late adulthood onset with foveal sparing, normal ff-ERG, and better visual prognosis is also recognized [4,15].

### 1.2. Genetics

**Figure 2:**
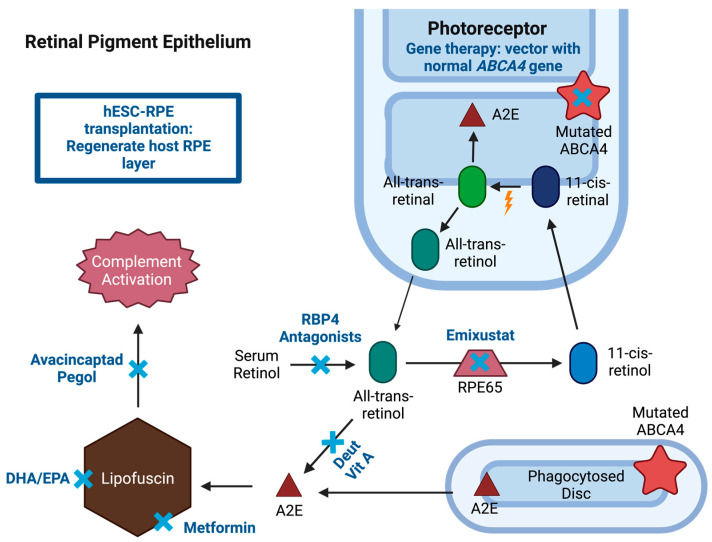
Potential pharmaceutical interventions for Stargardt disease (STGD1). Treatment options (blue “x”) are shown in a summarized representation of the visual cycle with mutated ATP-binding cassette, subfamily A, member 4 (*ABCA4*) gene resulting in the accumulation of lipofuscin in the retinal pigment epithelium (RPE). Treatment for STGD1 includes several strategies. Gene therapy includes vector delivery of human *ABCA4* gene. Pharmaceutical therapies include visual cycle modulators (VCM), metformin, avacincaptad pegol, and docosahexaenoic acid (DHA) and eicosapentaenoic acid (EPA). VCM represented are retinol binding protein 4 (RBP4) antagonists, deuterated vitamin A, and retinal pigment epithelium-specific 65 kDa protein (RPE65) inhibitor (emixustat). Stem cell therapy includes human pluripotent stem cell-derived retinal pigment epithelium (hESC-RPE) transplantation for regenerating RPE layer. Created with BioRender.com.

STGD1 is caused by an autosomal recessive mutation of *ABCA4* gene, located in the rim of outer segments of the photoreceptor cells and involved in the visual cycle. Photoreceptors during the visual cycle are constantly producing new disc membranes, pushing the older membranes further out towards the RPE [16]. One of the roles of the RPE is to phagocytose the most distal disc membranes with retinoid waste derivatives. ABCA4 proteins are “flippase” transporters, located in the outer segment disc membranes of rod and cone photoreceptors, which move retinoids from the lumen to the cytoplasmic side of the disc membranes mainly in the form of N-11-cis-retinylidene-phosphatidylethanolamine (NrPE) [8,17]. This allows the continuation of the visual cycle and decreases the production of bisretinoid compounds such as phosphatidyl-pyridium bisretinoid (A2PE) in the photoreceptor outer segment. As new disc membranes are produced, the older ones assume a more distal position, and the distal discs are phagocytosed by the RPE cells. After phagocytosis, the A2PE is converted to N-retinylidene-N retinylethanolamine (A2E) compound, which is insoluble and accumulates in the RPE cells to form lipofuscin (Figure 2) if it is not regularly managed by ABCA4 and other mechanisms [16]. With a mutated ABCA4, the accumulation of A2E and other bisretinoids leads to accumulation of toxic lipofuscin within the RPE, which with time will cause RPE cell death and photoreceptor dysfunction from a lack of RPE support [8,17]. The severity of phenotype correlates with the remaining ABCA4 function where more severe mutations, such as two null variants, result in a more severe phenotype with earlier onset and worse visual prognosis [13].

Other genes, such as elongation of very long chain fatty acids (*ELOVL4*) gene (STGD3) and prominin-1 (*PROM1*) gene (STGD4), produce autosomal dominant forms of the disease and are beyond the scope of this review [18,19]. The term STGD2 was discovered to be caused by the *ELOVL4* gene involved in STGD3 and was discontinued in 2005 [20].

## 2. Genetic Therapy

### 2.1. Introduction

Gene therapy has garnered a lot of attention, providing great potential in the realm of inherited retinal degenerative diseases. The main obstacle to the development of gene therapy for STGD1 is the large size of *ABCA4* gene (6.8 kb coding sequence) [21]. Gene therapy delivery systems include lentiviral vectors, adeno-associated viral (AAV) vectors, and non-viral vectors (Table 1A). Upcoming pre-clinical studies are evaluating non-viral delivery systems such as covalently closed and circular DNA (C3DNA) and nanoparticles [22,23]. These newer methods of delivery are posed to avoid inflammatory complications associated with viral vector delivery.

### 2.2. Lentiviral Vectors

The first phase I/II nonrandomized multicenter study (NCT01367444) of subretinal injection delivery of an Equine infectious anemia virus (EIAV)-based lentiviral vector carrying *ABCA4* (SAR422459, Sanofi) was terminated in 2019. Parker et al. recently published three-year follow-up data from the first five cohorts of 22 patients using three escalating doses of SAR422459 delivered to 12 patients subfoveally and 10 patients extrafoveally. Study outcomes included ocular and systemic adverse events as well as best corrected visual acuity (BCVA), kinetic perimetry, total full field of vision, ff-ERG, multifocal ERG, color fundus photography, FAF, and OCT [24]. The most common adverse events were associated with the surgical transvitreal subretinal injection technique, the most significant being the worsening of RPE atrophy [24]. Six (27%) patients developed worsening RPE atrophy on FAF imaging. One case of chronic ocular hypertension was reported. No patients had any clinically significant changes in their functional vision measurements. One patient demonstrated a significant decrease in the number of macular flecks in the treated eye compared to the untreated eye; however, ellipsoid zone (EZ) line loss and RPE atrophy quantification on OCT and functional vision parameters were similar between the two eyes. The study has some limitations including the small sample size and relatively short follow-up periods [24]. Despite these limitations, the study provides valuable insight into the safety of using subretinal injections to deliver gene therapy, yet potential long-term efficacy still needs to be determined. With early termination of the study, patients were transferred to a 15-year open-label safety study (NCT01736592) (Table 1A). The lack of functional improvements may also be associated with the lentiviral vector dimensions and structure, which may limit its transduction into the retina [25]. No other study involving lentivirus for STGD1 is ongoing in 2023.

### 2.3. Adeno-Associated Viral Vectors

AAV is a gene therapy delivery system that has shown safety with minimal adverse events in various trials; however, a major limitation of AAVs as vectors is their limited cargo capacity which is estimated to be around 4.7 kb [26]. Given the large coding region of the *ABCA4* gene (50 exons and 150 kb), several in vitro models have been studied. The first attempt included creating an “oversized” transgene with the complete 6.8kb coding sequence yet transduction of these genes did not lead to the production of full-length protein [27]. Another model includes dual vector AAV strategies which can include fusing overlapping gene regions to create a transgene by fragmenting the original gene size. An alternative technique involves trans-splicing which provides no overlap between two transgenes since repeated sequences are removed, leaving an intact full-length coding sequence. Pre-clinical studies have shown early signs of success in delivering full-length *ABCA4* coding regions using dual AAV and trans-splicing techniques (Figure 2) [28,29]. Given the positive outcomes obtained from animal studies, several studies are forthcoming using AAV as a gene therapy delivery system for individuals with STGD1.

AB0-504 (Abeona Therapeutics) is a dual AAV vector delivery system created by the Cre-LoxP recombinase system to reconstitute the full-length *ABCA4* gene and is currently being tested in pre-clinical models (Table 1A). Preliminary results presented at the 26th Annual Meeting of the American Society of Gene & Cell Therapy provided evidence of the dual AAV vector system’s capability to generate the complete ABCA4 protein in cell culture [30]. Recent proof-of-concept investigations have further expanded upon these results, showcasing the expression of both *ABCA4* messenger RNA (mRNA) and intact ABCA4 protein within the retinal tissue of ABCA4^−/−^ knockout mice following subretinal administration. Notably, the observed levels of *ABCA4* mRNA and full-length protein in the treated mice closely resemble those found in the naturally occurring ABCA4 of wild-type animals [30,31].

Moreover, other companies such as ViGeneron have also tapped into a novel dual AAV technology platform, achieving efficient expression of large genes in photoreceptors after intravitreal injection (Table 1A). They created VG-801 by using REconstitution Via mRNA Trans-splicing (REVeRT) technology enabling split genes encoding the 5′ and 3′ portions of human *ABCA4* to be packaged into dual vgAAV vectors, which led to the generation of full-length protein. Notably, the REVeRT technology demonstrated high efficiency in reconstituting both transcript and protein levels of ABCA4 [32].

OCU-410ST from Ocugen is using AAV5-hRORA, an AAV serotype 5 capsid-containing genetic material encoding human retinoic acid receptor-related orphan receptor alpha (RORA) as a possible treatment for *ABCA4*-associated pathologies (Table 1A). RORA is considered part of the nuclear hormone receptors modulating functions such as photoreceptor development, metabolism, phototransduction, and inflammation. Preliminary results presented at the 2023 The Association for Research in Vision and Ophthalmology (ARVO) Annual Meeting showed decreased autofluorescence values and higher scotopic b-wave amplitude with greater recovery post-photobleaching in ABCA4^−/−^ mice subretinally injected with AAV5-hRORA, indicating improved retinal function [33]. A phase 1/2 clinical trial using AAV5-hRORA is planned [34]. RTx-015 from Ray Therapeutics Inc. is also using an AAV vector for intravitreal delivery and pending clinical trials (Table 1A) [35,36].

The findings from these studies will contribute valuable knowledge to the field of gene therapy, specifically in the context of AAV-mediated transgene delivery, and may pave the way for potential treatment options for individuals with retinal degenerative conditions.

### 2.4. Optogenetics

Other companies are exploring the capability of AAV vectors to deliver genes that confer the inner retinal sensitivity to light bypassing degenerated photoreceptors altogether in end-stage Stargardt disease. In 2022, Nanoscope Therapeutics initiated a nonrandomized phase I/IIa clinical trial (NCT04919473) aimed at evaluating the safety and beginning to explore the efficacy of an intravitreal injection of an AAV viral vector carrying a multi-characteristic opsin-I (vMCO-I) gene which encodes a light-sensitive ion channel in patients with Stargardt disease due to *ABCA4* or other genes. The vector leads to the expression of the opsin in bipolar cells. When illuminated by ambient light, it leads to depolarization of bipolar cells conferring sensitivity to ambient light. An ongoing multicenter open-label phase II clinical trial (STARLIGHT, NCT05417126) is being conducted to evaluate the safety and efficacy of a single intravitreal injection of vMCO-010 in up to six subjects with STGD due to *ABCA4*, *ELOVL4*, or *PROM1* mutations and visual acuity worse than 20/640 (Table 1A). The company recently reported on preliminary results at the 2023 American Society of Retina Specialists meeting showing 3 dB gain in mean visual field perimetry and no serious adverse events [37,38].

### 2.5. Non-Viral Delivery via Covalently Closed and Circular DNA (C3DNA)

C3DNA is a non-viral gene therapy platform developed by Intergalactic Therapeutics in IG-002 that enables the delivery of large genes, does not integrate into the genome, allows for redosing, eliminates safety concerns associated with immune reactions to viral vectors, and allows for precise engineering using synthetic biology (Table 1A) [39]. The lead delivery modality for C3DNA therapies is cellular delivery of genetic material by Electro-Transfer (COMET) which combines a novel electrode design, custom wavelengths and algorithms, and an electrical field [40]. C3DNA does not use viral or bacterial sequences avoiding triggering immune reactions and potentially allowing redosing [40]. Recent data from preclinical studies provide novel evidence of sustained (12-month) expression of human ABCA4 protein in adult porcine retinas and 6-month expression of ABCA4 in non-human primate retinas following a single subretinal administration of a DNA payload encoding the *ABCA4* gene [41]. The company is planning future clinical development for patients afflicted with *ABCA4*-related retinopathies.

## 3. Pharmacological Therapies

### 3.1. Introduction

Emerging pharmacological therapies aim to reduce symptoms and inhibit the progression of STGD1 by targeting specific steps in the visual cycle that are altered by disease pathophysiology [42,43]. The visual cycle is a complex series of biochemical reactions that occur in the RPE and photoreceptor outer segments as part of the visual cycle, involving the primary photoreceptor molecule of vision composed of an opsin molecule and the chromophore 11-cis-retinal [44,45]. With light stimulation of the chromophore, 11-cis-retinal converts to all-trans-retinal, leading to the signal transduction cascade that allows the sensation of light [44,45]. The all-trans-retinal is then converted back to 11-cis-retinal through multiple steps in the outer segments and RPE cells involving numerous enzymes, one of which is the retinal pigment epithelium-specific 61 kDa protein (RPE65) [46]. ABCA4 transporters, found in the photoceptor outer segment disc membranes, are necessary for the elimination of all-trans-retinal from the disc lumens and recycling back to 11-cis-retinal. With *ABCA4* gene mutations found in STGD1, a nonfunctional ABCA4 protein hinders this removal of all-trans-retinal from photoreceptors, which leads to the production of A2PE within outer segments and buildup of A2E, a major component of toxic lipofuscin, in the RPE cells. This leads to degeneration of RPE cells and subsequent photoreceptor loss [16,17]. Although currently proposed pharmacological treatments for STGD1 are not curative, multiple studies and clinical trials have found promising success by targeting the key pathological pathways associated with STGD1 disease symptoms and progression.

### 3.2. RPE65 Enzyme Inhibition

Emixustat hydrochloride (ACU-4429, Kubota Vision Inc.) is an orally available visual cycle modulator that binds the RPE65 isomerase (Figure 2). Inhibiting the RPE65 enzyme depletes regeneration of visual chromophore 11-cis-retinal, which in turn reduces the production of all-trans-retinal [47]. Since these substrates are necessary for the production of bisretinoid components including A2E, this can reduce the accumulation of lipofuscin toxins in the RPE and decrease the rate of RPE and photoreceptor dysfunction and death [43]. In ABCA4^−/−^ mice with A2E accumulation in RPE, treatment with emixustat significantly reduced A2E accumulation when compared to controls [48].

Phase 1 clinical trials (NCT00703183 and NCT00942240) with healthy subjects showed that once-daily oral administration of emixustat was able to slow the rod visual cycle and was well tolerated, even in multiple doses up to 75 mg, with dose-dependent inhibition of b-wave rod responses on ERG [42]. The drug was originally developed for slowing the progression of geographic atrophy (GA) in age-related macular degeneration (AMD) but failed to show a significant difference in lesion growth rate for treatment compared to the placebo in phase 2 (NCT01002950) and phase 2b/3 (NCT01802866) clinical trials [49,50]. It is currently being assessed as a potential treatment for STGD1. A phase 2 trial (NCT03033108) was completed in 2017 in the US with 23 subjects treated with several doses of the drug for 1 month, which demonstrated emixustat biological activity with suppression of the visual cycle in STDG1 patients. This was shown with suppression of recovery in b-wave amplitude after photobleaching in rods. Rod b-wave amplitude of ERG represents the extent of rhodopsin regeneration with a proportional relationship between the magnitude of rod–wave amplitude and rhodopsin levels. The rate of recovery over time reflects the regeneration rate of rhodopsin levels and therefore 11-cis-retinal levels, which represents RPE65 activity [43]. Near complete suppression of b-wave amplitude recovery post-photobleaching was reported at 10 mg of emixustat. Few clinically significant findings were observed with a BCVA change from baseline of −11 to +9 letters on the Early Treatment Diabetic Retinopathy Study (ETDRS) VA chart. The main ocular side effects were delayed dark adaptation and dyschromatopsia with no serious adverse events reported [43]. A 24-month phase 3 trial (NCT03772665) using the 10 mg or placebo once daily, the dose selection from the phase 2 trial, was completed in 2022 (Table 1B). With 194 participants, the rate of macular atrophy growth as measured by FAF in STGD1 patients on 10 mg of emixustat was similar in comparison to that of placebo [51]. However, the preliminary post hoc analyses in a subgroup of 55 subjects with smaller atrophic lesions at baseline showed a 40.8% reduction of lesion progression compared to placebo at 24 months [51]. Inhibiting RPE65 during earlier stages of STGD1 may provide additional beneficial effects in disease progression.

### 3.3. Vitamin A Dimerization Inhibition

ALK-001 (Alkeus Pharmaceuticals) is a deuterated vitamin A molecule that inhibits vitamin A dimerization. In the visual cycle without functional ABCA4 protein, vitamin A derivative all-trans-retinal converts to toxic bisretinoids such as A2E found in lipofuscin (Figure 2) [52]. Accumulation of lipofuscin leads to RPE and photoreceptor degeneration in STGD1 [52]. Decreasing vitamin A levels has been shown to slow A2E biosynthesis and considered a potential therapeutic target. A deuterated vitamin A has C20 hydrogen atoms replaced with deuterium atoms forming C20–D3-Vitamin A. Unlike the C20 carbon–hydrogen bond, the C20–D3 bond is much more difficult to cleave, which inhibits vitamin A dimerization, limiting production of A2E [52]. In ABCA4^−/−^ mice, a diet containing C20–D3 compared to a diet with vitamin A led to reduction in A2E levels, lipofuscin deposition, and improved ERG, indicating improved eye function [53]. Dietary C20–D3 in ABCA4^−/−^ mice was also able to prevent development of disease phenotype with interruption of C20–D3 in diet leading to returned development of disease phenotype [54].

Given these positive preclinical results, a phase I clinical trial (NCT02230228) was completed with 40 healthy subjects showing safety and tolerability of oral ALK-001. An ongoing multicenter 24-month phase 2 clinical trial (TEASE, NCT02402660) in 50 patients receiving oral ALK-001 in late-stage STGD1 was initiated in 2015 with an open-label extension (NCT04239625) initiated in 2020 (Table 1B). Preliminary data were presented at the 2022 ARVO Annual Meeting reporting that with approximately 90% of vitamin A replaced with CD20–D3 Vitamin A, ALK-001, the growth rate of atrophic lesions was significantly slower (21–28% slower compared to the untreated arm) after 2 years [55]. No clinically significant changes in BCVA were found. The drug was well tolerated with no reported severe adverse reactions [55]. A phase 3 trial is being planned. Deuterated vitamin A molecules like ALK-001 play a role in the pathogenesis of STGD1 and may be a potential intervention even for late-stage STGD1.

### 3.4. RPB4 Antagonists

Retinol-binding protein 4 (RPB4) antagonists are being explored as potential therapeutic agents for STGD1. RPB4 has been shown to be involved in STGD1 and dry AMD pathophysiology through the toxicity of accumulated lipofuscin in the RPE [56,57]. RPB4 is the only plasma transport that carries retinol (vitamin A) to peripheral tissues from the liver. In particular, it carries retinol to the RPE, leading to the formation of bisretinoids. A2E is a bisretinoid component, which is associated with the toxicity of lipofuscin [56,58]. Therefore, reducing RBP4 levels has been explored as a therapy for managing STGD1 by modulating the visual cycle and reducing bisretinoid synthesis.

Fenretinide (Sirion Therapeutics) is a synthetic retinoid RPB4 antagonist, which binds to RPB4, preventing the binding of retinol and leading to the elimination of the RBP4–fenretinide complex and decreased retinol concentration in plasma and the eye [59]. In ABCA4^−/−^ mice, fenretinide caused a dose-dependent reduction in serum retinol and RBP4 with subsequent reductions in visual cycle retinoids and decreased A2E and lipofuscin accumulations in the RPE [59]. This drug has shown success in reducing serum RBP4 and retinol levels in a dose-dependent manner in a phase II trial for dry AMD (NCT00429936). In comparison to the placebo, patients with 300 mg of fenretinide showed a reduction in lesion growth and even the incidence of choroidal neovascularization; however, 20% of this cohort stopped treatment due to adverse effects including delayed dark adaptation and visual disturbance [60]. Due to its chemotherapeutic properties, fenretinide may lead to adverse events with its systemic apoptotic potential in many cells including RPE cells [61,62]. No trials have been initiated for STGD1 patients.

A1120 (ICR-14967, Stargazer Pharmaceuticals), STG-001 (Stargazer Pharmaceuticals), and tinlarebant (LBS-008, Belite Bio Inc.) are non-retinoid RPB4 antagonists. In ABCA4^−/−^ mice, A1120 reduced serum RBP4 and accumulation of lipofuscin bisretinoids; however, it did not suppress b-wave ERG recovery after photobleaching [56]. This may indicate that A1120 may not be able to produce sufficient biological activity with adequate suppression of the visual cycle in STGD1 patients [56]. Unlike fenretinide, A1120 is not a retinoic acid receptor-alpha agonist, which may improve safety and reduce adverse effects [56]. Clinical trials will be needed to determine A1120 efficacy in STGD1. For STG-001, a phase 1 trial (ACTRN12619000816156) for safety and tolerability was completed in 2019 in healthy patients. A subsequent phase 2 trial (NCT04489511) was completed in 2021 for the safety of once-daily oral STG-001 for 28 days in 10 subjects with STGD1 disease with no reported serious adverse events despite some reported visual disturbances (Table 1B). Similarly, a randomized, double-blinded, placebo-controlled phase 1 trial (NCT03735810) was completed in 2019 for safety and tolerability of tinlarebant in healthy adults aged 18–65 after single and multiple doses. Subsequently, a multicenter, randomized, double-masked, placebo-controlled phase 3 trial (NCT05244304) was recently initiated in 2023 for the treatment of STGD1 in adolescent subjects aged 12–20 (Table 1B). The primary outcome will be a change in atrophic lesion growth rate at 24 months for patients receiving 5 mg tinlarebant once a day orally in comparison to the placebo. Further developments will be necessary to determine if tinlarebant and other RPB4 antagonists will enable adequate suppression of the visual cycle and maintain an acceptable safety profile in STGD1 patients.

### 3.5. Complement C5 Inhibition

Avacincaptad pegol (Zimura, Inveric Bio, Granbury, NJ, USA) is a complement factor C5 inhibitor. Lipofuscin accumulation with increased bisretinoids including A2E in RPE can lead to complement activation (Figure 2) [63]. C5 complement is essential for the formation of an inflammasome and membrane attack complex in the final stage of the complement pathway, leading to cell death and activation of inflammatory responses [64]. Markers of complement activation were elevated in ABCA4^−/−^ mice, which led to basal laminar deposits within Bruch’s membrane due to lipofuscin accumulation and photoreceptor degeneration [65]. In the same mouse model, when a complement-negative regulatory protein was increased in the RPE, the bisretinoid accumulation was decreased in comparison to controls with decreased photoreceptor degeneration, indicating that inhibition of the complement system could be a therapeutic target for STGD1 [65].

Avacincaptad pegol is delivered by intravitreal injection and has been explored as a potential therapy for several disorders including STGD1, dry AMD, neovascular AMD, and idiopathic polypoidal choroidal vasculopathy (NCT03364153, NCT02686658, NCT03362190, NCT03374670). A randomized, double-masked phase 2/3 clinical trial (NCT02686658) was completed in 2019 with 286 subjects with geographic atrophy receiving intravitreal avacincaptad pegol. A significant reduction of 27% in geographic atrophy mean growth rate was observed in patients receiving either 2 or 4 mg doses versus sham over 12 months without serious adverse events [64]. Continued reductions in GA growth rate compared to sham were observed after 18 months in 2 mg and 4 mg cohorts, of 28% and 30%, respectively [66]. Unfortunately, macular neovascularization (MNV) was more frequent in the 2 mg (11.9%) and 4 mg (15.7%) cohorts than in their respective sham control groups (2.7% and 2.4%). A randomized, double-masked phase 2b trial (NCT03364153) was initiated in 2018 with an estimated enrollment of 120 subjects with STGD1 (Table 1B). The primary endpoint will be the mean change in the area of ellipsoid zone defect for patients receiving avacincaptad pegol compared to the sham dose group after 18 months. Due to the similar activation of the complement pathway between AMD and STGD1, a similar outcome with a reduction in atrophic growth rate may be possible for STGD1.

### 3.6. RPE Macroautophagy Stimulation

Metformin hydrochloride is a biguanide antidiabetic agent and is commonly prescribed oral medication for managing type 2 diabetes mellitus through suppressing liver gluconeogenesis and increasing peripheral insulin sensitivity (Figure 2) [67]. Metformin has also been reported to increase macroautophagy, essential for the degradation of cellular components damaged by reactive oxygen species, via the AMP-activated protein kinase (AMPK)-mammalian target of rapamycin (mTOR) pathway in many tissues [68]. AMPK maintains cellular energy homeostasis by regulating important processes including autophagy and can activate autophagy by inhibiting mTOR [69]. Interventions that increase stimulation of this pathway may improve RPE clearance of lipofuscin, which has the potential to decrease *ABCA4* retinopathy disease progression by decreasing lipofuscin accumulation, thus slowing RPE and photoreceptor degeneration [70,71]. Metformin was shown to enhance autophagy via the AMPK pathway in human retinal pigment epithelial cells in response to hydrogen peroxide-induced oxidative damage [69]. In ABCA4^−/−^ mice, oral metformin was shown to decrease lipid and lipofuscin accumulation in the RPE/choroid [72]. An ongoing open-label phase 1/2 trial (NCT04545736) was initiated in 2020 through the National Eye Institute with an estimated enrollment of 45 patients with *ABCA4* retinopathies including STGD1 (Table 1B). The trial aims to determine the safety and potential efficacy of up to 2000 mg of oral metformin for decreasing the rate of photoreceptor degeneration. This will be measured as the change in the growth rate of the square root transformed area of EZ band loss from baseline to 24 months. If successful, metformin may be an accessible and safe method for treating STGD1.

### 3.7. Omega-3 Fatty Acids

Docosahexaenoic acid (DHA) and eicosapentaenoic acid (EPA), types of omega-3 polyunsaturated fatty acids, have been investigated due to their role as components of photoreceptor cells. These polyunsaturated fatty acids are esterified into phospholipids and help maintain photoreceptor membrane fluidity, retinal integrity, and visual function [73,74,75]. DHA is mainly located in photoreceptor cells and plays an essential role in the synthesis of disc membranes, rhodopsin activation, and rod and cone development. Supplementation of dietary DHA significantly increases DHA in the brain and retina [76]. Omega-3 fatty acids in preclinical studies have shown the potential to generate anti-inflammatory mediators and resolvins and reduce complement 3 and lipofuscin levels (Figure 2) [73,77,78]. Furthermore, 24-month wild-type mice demonstrated reduced lipofuscin granule formation in the retina and produced a thicker outer nuclear layer when their blood ratio of arachidonic acid (AA) to EPA was <2, ideally 1–1.5, achieved by administration of omega-3 fatty acid supplementation [77].

A phase I clinical trial (NCT00060749) that ended in 2007 utilized DHA supplementation as a tool for macular function improvement in patients with STGD1. In this double-masked, randomized study with 11 participants, patients received either oral DHA supplementation with the dosing of 2000 mg per day or a placebo. However, unlike in preclinical mice studies, no improvement in macular function was observed [79]. Similarly, a non-randomized open-label study (NCT00420602) completed in 2017 that administrated 650 mg daily of DHA and 350 mg daily of EPA in STGD3 patients also did not attenuate the progression of maculopathy [80]. More recently, omega-3 fatty acids supplements (Eyetas) have also been investigated in patients with dry AMD and STGD1 with a prospective, randomized, double-blind study (NCT03297515) completed in 2020 (Table 1B) [81]. Preliminary results at 6 months with 21 patients showed a 6 ETDRS letter improvement in the omega-3 fatty acid group compared to the placebo group [81]. Omega-3 fatty acids may be beneficial supplements for managing patients with STGD1.

## 4. Stem Cell Therapy

### 4.1. Introduction

Several stem cell studies in animals and humans have been conducted or are currently being conducted to investigate stem cell therapy for STGD1 in an effort to repair retinal tissue and consequently improve VA. These trials mainly focus on the use of human embryonic stem cells (hESCs) [82].

### 4.2. Human Pluripotent Stem Cell-Based RPE

Preclinical studies that transplanted hESC-derived mature RPE cells into the subretinal space of eyes in the rat model of inherited retinal degeneration, which undergo visual deterioration due to a mutation in Mertk (a c-mer proto-oncogene kinase receptor specific to RPE cells), showed promising results [83,84]. Rats that received a subretinal injection of 5000 to 100,000 hESC-RPE cells with concomitant immunosuppression had significantly better VA by optomotor responses compared to untreated animals without teratoma production or other adverse events [84]. The success achieved in preclinical studies led to the development of several phase I/II clinical trials for STGD1 worldwide (NCT01345006, NCT01469832, NCT02445612, NCT01625559, NCT02941991, NCT02749734, and NCT02903576). Trials with reported findings are summarized (Figure 2).

The first phase I/II human clinical trials (NCT01345006, NCT01344993) completed in the United States in 2015 injected hESC-RPE subretinally in late-stage STGD1 patients and atrophic AMD, sponsored by the Astellas Institute for Regenerative Medicine [82,85]. Patients in different cohorts received a subretinal injection of 50,000 hESC-RPE cells, 100,000 hESC-RPE cells, and 150,000 hESC-RPE cells in suspension and received systemic immunosuppression to prevent rejection [82]. Preliminary data in one patient with STGD1 and a second patient with dry AMD showed no hyperproliferation, abnormal growth, or immune-mediated transplant rejection at 4 months after transplantation [85]. In the treated eye of the STGD1 patient, VA improved from hand motion to 20/800 and from 0 to 5 letters on the ETDRS VA chart. In the treated eye of the AMD patient, VA improved from 21 to 28 letters [85]. Final results were published on nine STGD1 patients and nine dry AMD patients showing that at the median follow-up of 22-month (4 patients < 12 months, 12 patients for 12–36 months, and 2 patients >36 months) treated eyes did not develop abnormal proliferation, obvious rejection, or serious ocular or systemic adverse events related to transplanted cells [82]. Adverse events were associated with the immunosuppressive systemic therapy required for the first 3 months after cell transplantation to reduce the risk of rejection. Thirteen out of 18 eyes demonstrated patches of increased subretinal pigmentation and BCVA improved in 10 treated eyes and deteriorated in one treated eye with no improvement found in untreated fellow eyes [82]. Vision-related quality-of-life measures increased for general and peripheral vision by 16–25 points 3–12 months post-transplantation in patients with AMD and by 8–20 points in patients with STGD1 [82]. A third phase I/II trial (NCT01469832) performed in the United Kingdom and sponsored by Astellas, in which 12 STGD1 patients were transplanted using the subretinal injection of 50,000 to 200,000 hESC-RPE cells, revealed no uncontrolled proliferation or inflammatory responses. No significant changes were seen in VA, microperimetry, or a quality-of-life questionnaire at 1-year follow-up [86].

The fourth phase I trial was completed in Korea (NCT01625559) and sponsored by CHA Biotech Co. It showed no serious adverse events for three patients with STGD1, 3 years after subretinal implantation of 50,000 hESC-RPE cells, with a similar protocol used in the other Astellas’ trials mentioned above [87]. One patient improved from a BCVA of 1 ETDRS letter to 10 ETDRS letters in the treated eye. The remaining patients maintained stable vision at 3 years [87]. Long-term follow-up of a minimum of 3 years in 13 STGD1 and 11 AMD patients treated in original phase I/II trials was presented at the 2019 ARVO annual meeting through the long-term follow-up studies (NCT02941991, NCT02445612) sponsored by Astellas (Table 1C) [88]. At 3 or more years after transplantation, there was no evidence of unanticipated persistent inflammation, hyperproliferation, tumor, or ectopic (non-RPE) growth. Patches of increased subretinal pigmentation were seen in 92% of eyes [88]. Treated eyes improved by a mean of 4.1 ETDRS letters versus 3.3 ETDRS letters in the untreated eyes at 3 years and BCVA was reported to improve most substantially within the first 2 years, with diminished improvements in the 3rd year [88,89]. With further research, hESC-RPE may prove to be a promising approach for the long-term treatment of STGD1. Patients should be educated and cautioned against participating in trials that do not follow the accepted norms of clinical trials. Patients should steer away from any trial that requires payment to participate, even if a trial is listed on clinicaltrials.gov, which is not reviewed or approved for safety and scientific integrity by the United States government or any other agency [90].

## 5. Conclusions and Future Directions

STGD1 is one of the most common inherited retinal degenerative diseases and is highly genetically heterogeneous [3]. No treatment is currently available. Clinical management of STGD1 typically involves the utilization of low vision aids and adaptive strategies to optimize visual function and enhance patients’ quality of life as well as instructing the patient to avoid vitamin A supplements and use sunglasses to protect their eyes from sunlight [91,92]. Multiple strategies have been explored for potential interventions, with many showing promise in preclinical studies and clinical trials. We summarize the current trials, completed and ongoing in 2023, for STGD1 involving gene therapy, pharmacological treatments, and stem cell therapy.

For gene therapy, SAR42259 was shown to have long-term safety and tolerability at 3 years with no significant functional changes, potentially indicating that the lentiviral vector may be limited for retinal transduction [24,25]. While the *ABCA4* gene was previously considered to be too large for AAV vectors [26], preclinical studies have shown success with delivering the gene through dual AAV vectors (ABO-504, VG-801) [28,29] and a non-viral delivery system, C3DNA (IG-002) [39,40,41]. Further studies will be needed to determine if these systems will be safe for humans and lead to changes in disease progression. AAV-based systems have also been used to deliver other gene products with potential for STGD1 intervention including vMCO-I (vMCO-010) [37], which confers photosensitivity to the inner retina bypassing degenerated photoreceptors in end-stage Stargardt disease, and RORA (OCU-410ST) [34], which supports photoreceptor function and homeostasis. vMCO-010 has shown success in improving functional vision in retinitis pigmentosa and has an ongoing trial for STGD [34]. While gene therapy requires invasive techniques like subretinal, suprachoroidal, or intravitreal injections, this potential intervention may provide long-lasting therapeutic effects. With the development of additional studies, gene therapy may prove to be a novel solution for the treatment of STGD1 and other retinal degenerative conditions.

In small molecule therapies, STGD1 intervention focuses on modulating the visual cycle, reducing A2E, and decreasing the effects of lipofuscin toxicity. Clinical trials for these interventions have shown variable results. Emixustat demonstrated suppression of the visual cycle with no significant BCVA changes in a phase 2 trial but failed to decrease the macular atrophy growth rate in STGD1 in the subsequent phase 3 trial with a large sample size [43,51]. ALK-001 shows some promise with preliminary data for the phase 2 trial demonstrating a decreased growth rate of STGD1 atrophic lesions after 2 years with no significant changes in BCVA [55]. Preliminary data for omega-3 fatty acid supplements (Eyetas) also indicate potential with 6 ETDRS letter improvement in BCVA for treated STGD1 and AMD patients compared to the placebo at 6 months [81]. Other potential pharmaceutical therapies including STG-001, tinlarebant, avacincaptad pegol, and metformin are being evaluated in ongoing clinical trials for treatment efficacy. While some of the small molecule therapies show potential for ameliorating the retinal degenerative effect of STGD1 with the lifelong use of oral medications or eye drops, additional studies with a larger number of patients and longer follow-up will be necessary to show their full therapeutic effect. Interventions may also be more effective in earlier stages of STGD1 with smaller atrophy sizes or before atrophy formation with permanent loss of RPE cells and photoreceptors. Alternative visual cycle modulators may also be explored for safe and effective interventions for STGD1.

Stem cell therapy may also be a viable avenue for STGD1 treatment. Numerous clinical trials have been performed with subretinal transplantation of hESC-RPE cells, which have shown long-term safety in STGD1 patients 3 years after implantation with limited BCVA improvement [88,89]. While this intervention may provide long-term protection and regeneration of the RPE, further studies are still necessary to study the effectiveness, duration, and continued safety of cell therapies in these patients. Further work is needed to ascertain these aspects of cellular therapy. Additional studies will also be necessary to determine the efficacy of stem cell therapy for functional improvement.

Overall, STGD1 is a complex, progressive disorder that leads to irreversible vision loss and disability without current treatment. Multiple treatment strategies have shown promise in attenuating disease progression. Further exploration will advance our understanding of underlying disease mechanisms and pave the way for the development of treatment solutions and interventions.

## Figures and Tables

**Table 1:** Recent Updates in Potential Stargardt Disease Interventions.

Treatment	Indication	Mechanism of Action	Sponsor	Stage	Results *
A. Genetic Therapy					
SAR422459 (EIAV-ABCA4)	STGD1	Lentiviral vector carrying normal coding sequence of human *ABCA4* cDNA	Sanofi, Paris France	Phase ½ (Initiated 2012) NCT01736592	No clinically significant vision changes or EZ line loss in 22 patients; six (27%) developed worsening RPE atrophy
ABO-504	STGD1	Dual AAV vector with full-length *ABCA4* gene	Abeona Therapeutics, Cleveland, OH, USA	Preclinical: ABCA4^−/−^ KO mice	Generate ABCA4 mRNA and protein in retinal tissue
VG-801	STGD1	Dual AAV vector for split genes encoding 5′ and 3′ parts of human *ABCA4*	ViGeneron, Planegg, Germany	Preclinical: Stargardt mouse model	General full-length ABCA4 protein
OCU-410ST	*ABCA4*-related retinopathies	AAV5-hRORA vector for retinal delivery of human RORA gene	Ocugen, Malvern, PA, USA	Preclinical, ABCA4^−/−^ KO mice, Phase 1/2 planned for 2023	Encode hRORA, decreased AF values and higher scotopic b-wave amplitude with greater recovery post-photobleaching
RTx-015	Retinitis pigmentosa, STGD	AAV vector	Ray Therapeutics, San Francisco, CA, USA	Preclinical	Unknown
MCO-010	STGD (due to *ABCA4*, *ELOVL4*, or *PROM* 1)	Optogenetics via AAV vector carrying ambient light activatable MCO protein gene expression cassette	Nanoscope Therapeutics Inc., Dallas, TX, USA	Phase 2a (Initiated 2022) NCT05417126	3 dB gain in mean visual field perimetry and no adverse events in 6 patients
IG-002	*ABCA4*-related retinopathies	Full-length *ABCA4* DNA with non-viral delivery through C3DNA platform	Intergalactic Therapeutics, Cambridge, MA, USA	Preclinical: in vitro RPE model	Sustained (12-month) expression of ABCA4 protein in adult porcine retinas following a single sub-retinal administration
B. Pharmacological					
Emixustat Hydrochloride	STGD1	RPE65 enzyme inhibitor to decrease formation of 11-cis-retinal, which ultimately reduces the accumulation of lipofuscin in RPE	Kubota Vision Inc., Tokyo, Japan	Phase 3 (Completed 2022) NCT03772665	194 subjects showed no change in macular atrophy growth, post hoc analyses in subgroup of 55 with smaller atrophic lesions at baseline showed 40.8% reduction in lesion progression after 2 years
ALK-001	STGD1	Deuterated vitamin A molecule that inhibits vitamin A dimerization	Alkeus Pharmaceuticals, Inc., Cambridge, MA, USA	Phase 2 (Initiated 2020) NCT04239625	Atrophic lesion growth rate significantly slower (21–28% decrease) in 50 patients after 2 years; no clinically significant changes in BCVA
STG-001	STGD1	Antagonist of serum non-retinoid RPB4	Stargazer Pharmaceuticals, Inc., Boston, MA, USA	Phase 2a (Completed 2021) NCT04489511	No reported serious adverse events despite some reported visual disturbances in 10 subjects
Tinlarebant	STGD1, dry AMD	Antagonist of serum non-retinoid RPB4	Belite Bio, Inc., San Diego, CA, USA	Phase 3 (Initiated 2022) NCT05244304	Safety and tolerability of after single and multiple doses in completed Phase 2
Avacincaptad pegol	STGD1	Complement factor C5 inhibitor	IVERIC bio, Inc., Cranbury, NJ, USA	Phase 2b (Initiated 2018) NCT03364153	286 subjects with GA showed reduction of 27% in GA mean growth rate; however, MNV was more frequent in (11.9–15.7%) for completed Phase 2/3 for AMD
Metformin hydrochloride	*ABCA4* Retinopathy	Increase macroautophagy via mTORC1/AMPK pathway of RPE	National Eye Institute (NEI), Bethesda, MD, USA	Phase ½ (Initiated 2020) NCT04545736	Decreased lipid and lipofuscin accumulation in RPE/choroid for ABCA4^−/−^ mice
Omega-3 Fatty Acids, DHA/EPA	Dry AMD, STGD1	Generates anti-inflammatory mediators, limit the accumulation of A2E	Ophthalmos Research and Education Institute, Nicosia, Cyprus	Prospective Trial (Completed 2020) NCT03297515	Six ETDRS letter improvement in 21 patients
C. Stem Cell Therapy					
hESC-RPE	STGD1, AMD	Sub-retinal transplantation of hESC-derived mature RPE cells in suspension to replace/improve health of the host RPE layer	Astellas Institute for Regenerative Medicine, Westborough, MA, USA	Phase 1/2 (Completed 2019) NCT02445612, NCT02941991	Patches of increased subretinal pigmentation in 92% of eyes with a mean of 4.1 ETDRS letters versus 3.3 ETDRS letters in the untreated eyes at 3 years for 13 STGD1 and 11 AMD patients

* Includes preliminary results and most updated associated results, STGD: Stargardt disease; AMD: age-related macular degeneration; GA: geographic atrophy; hESC-RPE: human pluripotent stem cell-based retinal pigment epithelium; MCO: multi-characteristic opsin; AAV: adeno-associated virus; *ABCA4*: ATP-binding cassette, subfamily A, member 4; RORA: RAR-related orphan receptor A; RPE65: retinal pigment epithelium-specific 65 kDa protein; A2E: N-retinylidene-N retinylethanolamine; mTORC1/AMPK: AMP-activated protein kinase/mammalian target of rapamycin complex 1; DHA/EPA: docosahexaenoic acid/eicosapentaenoic acid.

## Data Availability

No new data were created or analyzed in this study. Data sharing is not applicable to this article.
